# Induction of hypoxia and necrosis in multicellular tumor spheroids is associated with resistance to chemotherapy treatment

**DOI:** 10.18632/oncotarget.13857

**Published:** 2016-12-10

**Authors:** Silvio Däster, Nunzia Amatruda, Diego Calabrese, Robert Ivanek, Eleonora Turrini, Raoul A. Droeser, Paul Zajac, Carmela Fimognari, Giulio C. Spagnoli, Giandomenica Iezzi, Valentina Mele, Manuele G. Muraro

**Affiliations:** ^1^ Department of Surgery, University Hospital Basel, Basel, Switzerland; ^2^ Department of Biomedicine, University of Basel, Basel, Switzerland; ^3^ Department for Life Quality Studies, University of Bologna, Rimini, Italy

**Keywords:** multicellular tumor spheroids, three-dimensional culture, tumor model, hypoxia, necrosis

## Abstract

Culture of cancerous cells in standard monolayer conditions poorly mirrors growth in three-dimensional architectures typically observed in a wide majority of cancers of different histological origin. Multicellular tumor spheroid (MCTS) culture models were developed to mimic these features. However, *in vivo* tumor growth is also characterized by the presence of ischemic and necrotic areas generated by oxygenation gradients and differential access to nutrients. Hypoxia and necrosis play key roles in tumor progression and resistance to treatment. To provide *in vitro* models recapitulating these events in highly controlled and standardized conditions, we have generated colorectal cancer (CRC) cell spheroids of different sizes and analyzed their gene expression profiles and sensitivity to treatment with 5FU, currently used in therapeutic protocols. Here we identify three MCTS stages, corresponding to defined spheroid sizes, characterized by normoxia, hypoxia, and hypoxia plus necrosis, respectively. Importantly, we show that MCTS including both hypoxic and necrotic areas most closely mimic gene expression profiles of *in vivo*-developing tumors and display the highest resistance to 5FU. Taken together, our data indicate that MCTS may mimic *in vitro* generation of ischemic and necrotic areas in highly standardized and controlled conditions, thereby qualifying as relevant models for drug screening purposes.

## INTRODUCTION

Screening of novel anti-cancer agents is usually performed on cells from the US National Cancer Institute (NCI) 60 panel, a collection of established tumor cell lines representing nine distinct human tumor types [1], cultured in two-dimensional (2D) monolayers. These cells are easy to propagate and amenable to high-throughput studies.

However, cells growing in 2D inadequately reflect tumor growth, inasmuch as they lack features of the three-dimensional (3D) microenvironment, which deeply influence cell proliferation, differentiation, migration, and intracellular signal transduction *in vivo* [2–8]. Importantly, 3D architecture of tumor growth *in vivo* results in the generation of a variety of physical and chemical gradients contributing to zonation and phenotypic heterogeneity within the tumor [9–11]. In particular, *in vivo* oxygenation in tumors fluctuates temporally and regionally, as indicated by heterogeneous local partial oxygen pressure measured in mouse tumor xenografts [12, 13]. Fluctuations of hypoxia and re-oxygenation differentially influence tumor cells and have broad ranging implications for tumor gene expression profiles, progression, stress response, and signal transduction [14–16]. Resistance to radiotherapy or chemotherapeutic agents such as cisplatin or doxorubicin, has been suggested to be frequently associated with hypoxia [4, 17–23].

Based on this background, 3D cultures have been developed to bridge the gap between conventional 2D models and *in vivo* studies [24]. The multicellular tumor spheroid (MCTS) model, one of the best-established 3D culture methods, has been used for decades with proven superiority over monolayer cell culture models to recapitulate *in vivo* tumor growth [25].

Similarly to *in vivo* tumors, MCTS include hypoxic and apoptotic/necrotic areas, developing as a consequence of the formation of oxygen and nutrient gradients [26]. Remarkably, in MCTS hypoxia occurs gradually over time, upon increase of spheroid size. Early studies indicate that small micro-spheroids of <200μm diameter mostly include proliferating and normoxic cells [27, 28]. However, further growth to diameters of approximately 200-300μm results in a typical zonation, with proliferative zones at the surface co-existing with normoxic quiescent zones in the middle and hypoxic zones in the core [29]. Finally, in spheroids of approximately 500μm diameter, formation of necrotic areas is observed.

The use of MCTS for drug screening purposes is currently under evaluation. However, the possible impact of their different composition, with regard to hypoxic/necrotic areas, on tumor cell's drug response, has not been investigated in detail yet.

In this study, we exploited MCTS features to develop a culture system allowing the assessment, under highly controlled and standardized conditions, of the impact of spatial and temporal changes in oxygen levels on human colorectal cancer (CRC) cells.

Using CRC cells from established cell lines, we generated MCTS of progressively larger size and analyzed their gene expression profiles in comparison to that of *in vivo* generated tumors, and their sensitivity to current drug treatment.

## RESULTS

### Definition of MCTS maturation stages and histological characterization

**Figure 1:**
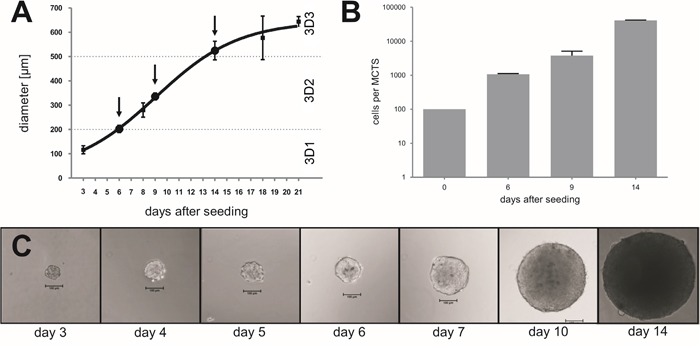
Establishment of CRC cell MCTS at different stages CRC cells from the HT29 cell line were initially seeded at 100 cells per hanging drop and cultured for the indicated time span. MCTS sizes were measured **A.** and cell numbers were counted **B.**, after trypsinization using the Neuebauer chamber, at the indicated time points. **C.** Representative pictures displaying progressive increase of MCTS size during culture. Magnification 10x; scale bar 100μm.

In initial studies we tested the growth kinetics of MCTS generated by different numbers of cells (100, 500, and 1000 cells per well), in order to select conditions resulting in the rapid generation of compact MCTS (data not shown). An initial density of 100 tumor cells per hanging drop optimally allowed, for both HT29 and HCT116 cells, the development of progressively growing spheroids (Figure 1 and Supplementary Figure S1). In keeping with previous studies [15, 30, 31], tumor cells cultured in 3D structures were characterized by slow proliferation reaching a plateau at 20 days (Figure 1 and Supplementary Figure S1).

**Figure 2:**
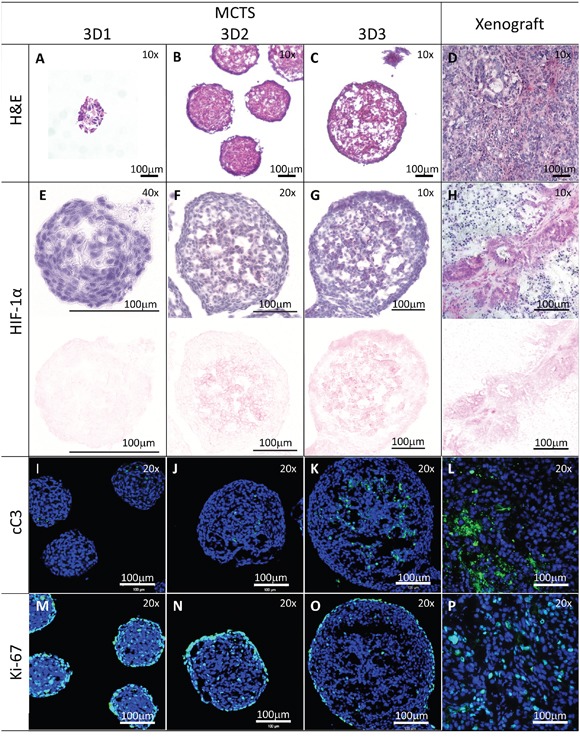
Histological characterization of MCTS of different sizes Sections from HT29 MCTS at the indicated stages or from xenografts generated in immunodeficient mice were stained with H&E panels **A-D.** or stained with anti-HIF-1α (panels **E-H.**; upper panel HIF-1α and counterstaining with Hematoxylin, lower panel color deconvolution to show the HIF-1α positivity only), anti-cleaved Caspase 3 (cC3, panels **I-L.**, green) or anti-Ki-67 antibodies (panels **M-P.**, green). Sections from panels I-P were counterstained with DAPI (blue). Panels A-D, G, H: magnification 10x; panels F, G, I-P: magnification 20x; panel E: magnification 40x. Scale bar 100μm.

We then analyzed MCTS collected at different time points, aiming at the identification of growth stages characterized by specific metabolic features. Indeed, previous studies reported that a hypoxic core begins to be formed in spheroids larger than 200μm and that a necrotic core is detectable in MCTS larger than 500μm [26]. In our culture conditions, we could collect MCTS with a diameter <200μm, thereafter referred to as 3D1 stage, composed on average of 1000 cells after 6-7 days, a diameter of 300-350μm, thereafter referred to as 3D2 stage, containing on average of 10000 cells after 9-10 days and a diameter of >500μm, thereafter referred to as 3D3 stage, with an average of 40000 cells after 14-15 days, as exemplified for HT29 cells in Figure 1. Interestingly, 3D2-3, but not 3D1 stage MCTS or cells cultured in 2D, displayed expression of hypoxia-inducible factor 1α (HIF-1α) protein, typically detectable in hypoxic areas [32], similar to tumor xenografts generated *in vivo* upon s.c. injection of the same cells in immunodeficient animals (Figure 2E-2H and Supplementary Figure S2). The expression of cleaved caspase 3 (cC3), known to be associated to apoptotic and necrotic events [33, 34] was undetectable in 3D1-2 stages MCTS and in cells cultured in 2D. In contrast, cC3 positive cells and apoptotic/necrotic cores were clearly visible in 3D3 stage as well as in xenografts (Figure 2K-2L). On the other hand, analysis of Ki-67 expression, associated with cell proliferation, revealed a highly compact and organized cell growth in outer MCTS layers at all maturation stages (Figure 2M-2O). However, in 3D1 stage proliferating cells were more abundant and were also present in the inner parts of the MCTS (Figure 2M). Similarly, in xenografts, Ki-67+ cells were detectable throughout the whole tissue sections and in the monolayer of cells cultured in 2D (Figure 2P and Supplementary Figure S2). Ezrin-radixin moesin-binding phosphoprotein 50 (EBP50) is a protein expressed in the apical portion of polarized epithelial cells and typically detectable in colon tissues. Specific staining suggested that HT29 tumor spheroids are characterized by organized internal structures, resembling those detectable in the differentiated epithelium of normal colon mucosa (Supplementary Figure S3).

### Gene expression analysis of cells cultured in spheroids of different size

**Figure 3:**
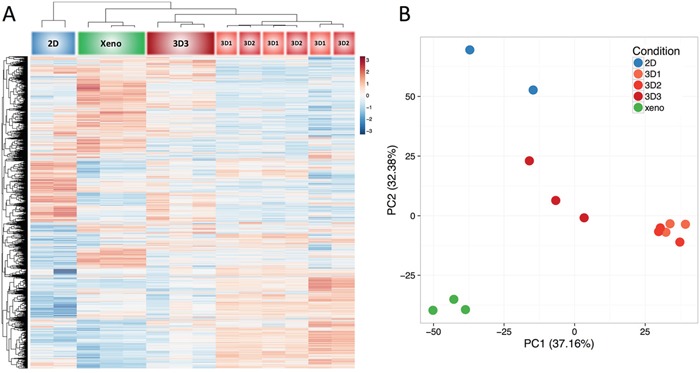
Gene Expression Analysis Total cellular RNA from HT29 cells cultured in 2D, MCTS at different stages, or as xenografts in immunodeficient mice, was purified and reverse transcribed. Next generation sequencing (NGS) was then performed. Results were evaluated by unsupervised clustering analysis **A.**, or Principal Component Analysis **B.** using 50% of most variable genes in the dataset (approx. 9000 genes).

Taking advantage of NGS technology, we compared gene expression profiles of cells cultured in monolayers (2D), as MCTS at different stages (see above), and growing as xenografts in immunodeficient mice (Xeno). An unsupervised hierarchical clustering, based on correlation of expression patterns, showed that the three-dimensional culture models clustered together with the corresponding xenografts (Figure 3A).

Strikingly, at difference with cells in monolayers, all MCTS cultures clustered together with corresponding xenografts (Figure 3A), thus suggesting that MCTS better mimic gene expression profiles of tumor cells *in vivo*. Interestingly, the 3D3 subset appeared to cluster most closely with xenograft-derived specimens, as compared to 3D1 and 3D2 cultures which, instead, clustered together.

We then evaluated the relationship between the groups (2D, 3D1, 3D2, 3D3, and Xeno) by performing a principal component analysis and plotting the samples along first two components which explained a majority of the variance present in the dataset (PC1 37.16%, PC2 32.38%) (Figure 3B). Consistent with cluster analysis data, 2D cultures and Xeno tissues were clearly positioned in coordinates different from each other, while 3D cultures showed an intermediate position between these conditions, with 3D3 MCTS being more close to xenografts than 3D1 or 3D2 (Figure 3B).

**Figure 4:**
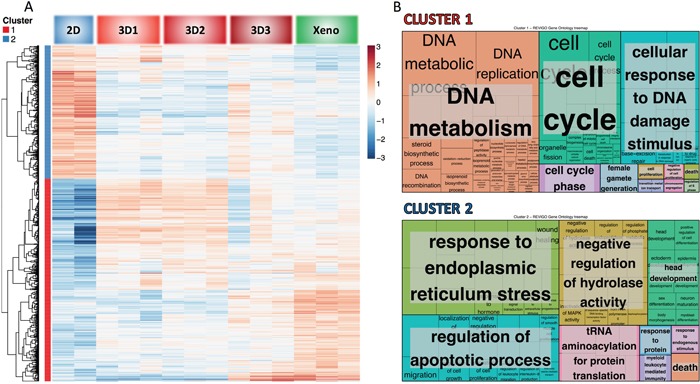
Clustering and functional analysis of genes Next generation sequencing (NGS) was performed on RNA from cells cultured in 2D, in 3D at the different stages (3D1, 3D2, 3D3) and grown as xenograft in mice. The most differently expressed genes in 2D and Xeno conditions were selected and among these genes we chose those more similarly expressed in 3D3 and Xeno conditions to generate the heatmap **A.** and the Treemap **B.** by using REVIGO. A Two major hierarchical clusters are identified. B The Treemap shows the most represented pathways in the GO term list. Each rectangle represents a single cluster of genes. Rectangles are then joined into smaller rectangles representing sub-branches, marked with different colors. Sizes of the rectangles are proportional to the p-value.

To investigate gene profiles' similarities in 3D3 cultures and xenografts, we first selected genes with the highest difference between 2D and Xenografts, of putatively higher relevance for “*in vivo*” tumor growth. Then, among genes differentially expressed in 2D and xenografts, we identified those with the highest similarity between 3D3 and Xenograft. A cluster analysis was then performed (Figure 4A and Supplementary File S1), using “DAVID” algorithm and “GO” as functional enrichment analysis [35]. Next, we ran the REVIGO analysis in order to identify which gene ontology categories showed similar expression in 3D3 and xenograft samples. For that purpose we limited the selection of genes to those which were differentially expressed in 2D and Xeno conditions and at the same time had similar expression levels in 3D3 and Xeno conditions. Hierarchical clustering revealed two major gene clusters, as indicated in the heatmap (Figure 4A).

The Treemap (Figure 4B) displays a two-level hierarchy of GO terms as a set of nested rectangles. Each box is representative of a single cluster. Each branch of the tree is given a rectangle, which is then tiled with smaller rectangles representing sub-branches. The Treemap shows the most represented pathways in the GO term list (Supplementary File S1). Sizes of the rectangles are proportional to the frequency of the GO terms in the database and they are colored to mark separate entities.

Cluster 1 is strongly downregulated in the 2D and upregulated in 3D3 and Xeno conditions. It comprises genes involved in DNA metabolism, cell cycle and response to DNA damage. These data underline the differential proliferative and mitogenic potential of cells growing in 3D and *in vivo*, as compared to the 2D culture.

Instead, cluster 2 is mainly upregulated in 2D as compared to 3D and Xeno and it includes genes involved in the response to endoplasmic reticulum (ER) stress and in the regulation of apoptotic process. Cell stress response, initially aimed at compensating for damage, is known to trigger cell apoptosis and death if ER dysfunction is severe or prolonged [36].

Considering the results observed in this analysis of the gene expression profile, we believe that the size of the spheroid has to be taken into account in the planning phase of specific drug testing, accordingly to the purpose. In addition, the 3D3 might be considered a good option for drug screening assay to mimic the colorectal cancer tissue where hypoxia and necrosis usually occur.

### Response to drug treatment in MCTS at different stages and monolayer cultures

Cells cultured in 3D structures have been shown to be characterized by a relative resistance to drugs included in current chemotherapy protocols [37]. Therefore, the use of MCTS, rather than cell monolayers, has been proposed for the screening of innovative anti-cancer compounds [38]. Indeed, hypoxia has been suggested to represent an important mechanism underlying cancer cell resistance to treatment [39]. Since MCTS size appears to be associated with hypoxia induction, we grew curious to evaluate the sensitivity to anti-cancer drugs of cancer cells cultured in different 3D conditions.

Considering the results described above, that show a similar gene expression profile between 3D3 and Xeno conditions in particular related to cell cycle, DNA metabolism, and apoptosis we selected 5FU as the most common anti-proliferative drug used for CRC treatment.

**Figure 5:**
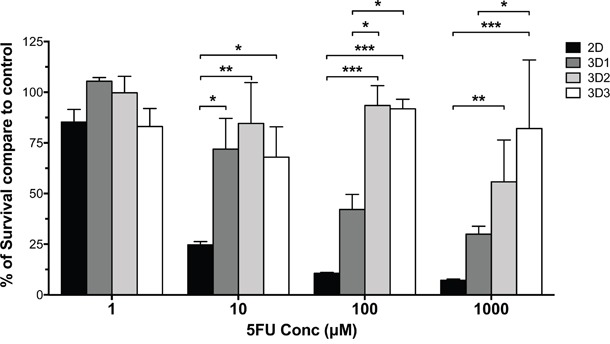
Sensitivity to drug treatment of HT29 cells cultured in monolayers or MCTS of different size HT29 cells cultured in monolayers or in MCTS at different stages were exposed to the indicated 5FU concentrations for three days. Percentages of viable cells were then evaluated, as compared to untreated cells cultured in the same conditions, by using a modified acid phosphatase assay.

At the lowest 5FU concentration (1μM), cells cultured in 2D or 3D at different stages displayed similarly high survival (>75%), irrespective of culture conditions. However, at 10μM we observed a significantly higher survival in cancer cells cultured in 3D, as compared to 2D, irrespective of MCTS stage (P<0.05). Most interestingly, at higher 5FU concentration (≥100μM), we could detect a significantly higher survival of cells cultured in 3D2 and 3D3 MCTS stages, as compared to those at 3D1 stage or 2D. Remarkably, in 3D3 stage spheroids cell survival exceeded 70% even in the presence of the highest 5FU concentration (1000μM) (Figure 5). Thus, MCTS stages characterized by lower numbers of Ki-67+ cells and HIF-1α and cC3 positivity appear to be associated with differential sensitivity to 5FU treatment.

Resistance to 5FU has been suggested to be associated with high expression of thymidylate synthase (TYMS) and thymidine kinase (TK) in *in vitro* experimental settings and in clinical specimens [40, 41]. Based on this background, we analyzed the expression of these enzymes at the protein level in HT29 cells cultured in monolayers or as MCTS at different stages. We observed a minor, non-significant (*P*:0.7) increase of TYMS expression in stage 3D1 spheroids, as compared with monolayer cultures, whereas TK expression was unmodified (Supplementary Figure S4). Surprisingly however, MCTS at the 3D2 and 3D3 stages, characterized by high resistance to 5FU treatment, displayed expression of TYMS and TK comparable to those detectable in bi-dimensional HT29 cultures, thus suggesting that additional mechanisms may underlie 5FU resistance in cells cultured in these conditions.

## DISCUSSION

Two-dimensional cultures of tumor cell lines are simple, convenient, and easily amenable to high-throughput drug screening studies [4, 42]. However, they fail to capture the complexity of tumor tissue structures *in vivo*, including the variable physical and chemical parameters promoting generation of hypoxic/necrotic areas and phenotypic heterogeneity of cancer cells [9]. 3D-culture models have been suggested to more reliably mirror the architecture of solid tumors and to represent important tools for the development of innovative *in vitro* assays and models of neoplastic cell growth with high potential clinical relevance. Indeed, cells cultured in 3D conditions have been shown to be endowed with specific characteristics, including resistance to apoptosis, and chemo- and radio-therapy, closely matching those frequently observed *in vivo* [2, 6, 15, 25, 43].

Tumor cell proliferation in avascular tumor nodules, micrometastases or inter-capillary microregions of solid tumors *in vivo*, generates gradients of concentration of nutrients, oxygen, and catabolites [44]. These conditions, combined with cell-to-cell and cell-to-matrix interactions occurring in three-dimensional structures, do impact gene and protein expression profiles and distribution and penetration of soluble factors, including drugs, possibly resulting in poor response to treatment, as typically described in avascular tumors where a formation of necrotic areas occurs [4, 5, 26, 31].

To investigate these phenomena, we have addressed the generation *in vitro* of 3D structures mimicking the sequential development of ischemic and necrotic areas within solid cancers. To reproducibly generate homogenous spheroids of uniform size, we cultured CRC cell from established cell lines in hanging-drops in multiwell plate format. In this model both HT29 and HCT116 cell lines displayed a slow, exponential growth kinetic for 21 days of culture. We classified the resulting MCTS in three growth stages. Upon Ki-67-specific staining, we observed that in the first stage proliferating cells are numerous and widely distributed within the MCTS whereas in the second stage they are mainly localized in the outer layer of the spheroid. Instead, in the third stage, proliferating cells are only detectable in a compact ring localized in the outer MCTS layer while inner cores are characterized by live quiescent cells or dying cells. On the other hand, HIF-1α and cC3 specific staining is only detectable in inner areas of larger size 3D2 and 3D3 MCTS. Notably, Ki-67, HIF-1α and cC3 specific staining in xenografts appears to present a different pattern, as compared to MCTS. Intratumoral vascularization, present in xenografts, but not in MCTS, is likely playing a major role in the generation of more heterogeneous differential staining for these markers.

Similarly to *in vivo* tumors, and consistent with previous data, in our system we found that the inner parts of HT29 MCTS with a >200μm diameter presented hypoxic areas, as indicated by HIF-1α-specific staining [26]. Moreover, by cleaved caspase 3-specific staining we could observe apoptotic/necrotic areas in the inner core of MCTS with a >500μm diameter (Figure 2). Most importantly, significantly different gene expression profiles were detectable in tumor cells cultured in monolayers or in the different 3D stages or growing in xenografts in immunodeficient animals. In particular, MCTS with a >500μm diameter, containing important physical parameters as hypoxia and necrosis, showed the highest similarity of gene expression profiles to murine xenografts. Notably, these similarities appeared to relate to biological processes involving cell cycle and apoptosis, which might have a direct consequence on treatment effectiveness.

Several studies in the past have shown that numerous genes are differentially expressed in cells growing as MCTS, as compared to monolayer cultures [45–50]. Indeed, hypoxia and necrosis not only directly regulate radio- and drug-resistance, but also indirectly modulate specific gene expression patterns, as is the case of HIF-1α-regulated genes. Also, culture of breast cancer or melanoma cells as MCTS has been shown to result in down-regulation of several DNA mismatch-repair genes, an effect that is also directly induced by hypoxia and/or necrosis [38].

In our study we did not observe obvious correlations between spheroid sizes and expression of hypoxia related genes. It should be considered that MCTS are characterized by an outer layer of proliferating cells while gradients of oxygen tension, nutrients and catabolites become increasingly challenging as the distance from the surface of the spheroid increases. Considering that gene expression analysis was performed on RNA from whole spheroids, it is conceivable that, similarly to solid tumors “*in vivo*”, heterogeneous physiological conditions within the MCTS drive the expression of genes involved in a multiplicity of biologically important processes, thereby “masking” events occurring in defined areas, which, in contrast, are detectable at the protein level, by immunohistochemistry.

To validate the potential clinical relevance of this model, we addressed the sensitivity of tumor cells in the different MCTS stages to drug treatment by using doses reflecting the clinical situation, where peak plasma concentrations of 300-500μM are detectable after a single injection of 5FU [51]. In keeping with previous reports [20, 52, 53], we observed that development of resistance to 5FU treatment is typically associated with the detection of ischemic areas within MCTS, whereas progress toward the development of necrotic areas within MTCS appears to associate with increasing resistance to treatment only at the highest dose tested. Accordingly, sensitivity to 5FU is associated to the presence of high percentages of Ki-67 proliferating cells in “early” stage MCTS.

Gene expression profile analysis, identifying gene clusters differentially regulated in cells cultured in different conditions, and showing differential sensitivity to 5FU treatment, provides an important database paving the way towards further investigations addressing the characterization of specific “resistance” markers of potential clinical relevance.

Resistance to 5FU may be attributable to poor diffusion within inner MCTS cores, similarly concerning oxygen, nutrients and drugs. However, surprisingly, a minor, non-significant increase of the expression TYMS gene, which has been associated with resistance to 5FU treatment, was only detectable in 3D1 MCTS, characterized by a relative sensitivity of tumor cells to 5FU. TK expression was similar in all culture conditions. It is thus tempting to speculate that the detection of ischemic areas within tumor tissues might represent a more reliable marker of resistance to 5FU treatment than increased TYMS or TK expression levels. Thus, 3D cultures including hypoxic areas, such as 3D2 and 3D3 MCTS, may uniquely allow the identification of additional drug resistance mechanisms, thereby qualifying as relevant models for drug screening.

Our study presents a number of obvious limitations. In particular, non-transformed cells present in tumor microenvironment, or anatomical location of cancers may impact on the sensitivity of tumor cells to drug treatment [54]. Moreover, patient to patient variations should also be considered. In these respects, the MCTS model presented here is obviously oversimplified.

Nevertheless, this *in vitro* model is amenable to larger scale drug screening procedures, allowing the testing of the effectiveness of defined compounds, or their combinations in highly controlled conditions, while reflecting specific microenvironmental conditions of potentially high clinical relevance.

Furthermore, it might be of interest in basic cancer biology studies, since it would allow, in controlled conditions, an accurate analysis of the molecular events inherent in the development of ischemic and necrotic areas within tumor micronodules in the absence of the confounding factors frequently complicating *in vivo* models.

## MATERIALS AND METHODS

### Cell cultures

Established human CRC cell lines HT29 and HCT116 were purchased from American Type Culture Collection (ATCC, Manassas, VA). HT29 was maintained in McCoy's 5A (Sigma-Aldrich) supplemented with 10% FBS and GlutaMAX-I (2mM L-alanyl-L-glutamine dipeptide), and with 10μg/mL kanamycin sulphate (Gibco). HCT116 cells were cultured in RPMI 1640 (Sigma-Aldrich) containing the same supplements plus HEPES, non-essentials aminoacids, and sodium pyruvate (Gibco). Cells were cultured at 37°C in a 5% CO_2_ incubator. Absence of mycoplasma contamination was verified by PCR testing prior to investigations.

### Generation of MCTS

MCTS were formed by the hanging drop method, using 96-well GravityPlus plates (Insphero AG, Schlieren, Switzerland) [25]. Briefly, cells were seeded as 40μL per well by top-loading. After seeding, plates were cultured at 37°C with 5% CO_2_ for 3–4 days to allow cell assembly for gravity-enforced and the formation of the MCTS. Half of culture medium was replaced every three to four days. All steps were performed using an automatic multichannel pipette at a flow rate of 10μL/s (Viaflo, Integra Biosciences, Zizers, Switzerland). Following microtissue formation, MCTS were transferred in the GravityTrap recipient plate (Insphero AG, Schlieren, Switzerland) allowing monitoring of the spheroid growth.

### Growth kinetics analysis

MCTS growth kinetic was examined at different time points using an inverted phase contrast microscope (Nikon Eclipse TS100, Nikon Co.) equipped with a digital camera (Nikon Digital Sight DS-2MBWc, Nikon Co.). To measure MCTS diameters, five pictures were acquired for each time point (Image-Pro Plus v4.5.1, Media Cybernetics). To count cells contained in MCTS at each of the three stages, spheroids from an entire 96 well GravityTrap recipient plate were collected at each time point, washed with PBS, and digested in Trypsin for 15 minutes at 37°C. Eventually remaining clusters were disrupted by pipetting. Recovered cells were counted using a Neubauer chamber and average number of cells per spheroid was calculated.

### Histological analysis

MCTS were harvested and fixed in cold methanol for 10 minutes at −20°C, washed in PBS, transferred to molds, embedded in OCT, and stored at −80°C until sectioning. Serial frozen sections were cut at 10μm with a cryostat, and mounted onto Superfrost Plus microscope glass slides (Menzel-Glaeser, Braunschweig, Germany). Sections were stored at −20°C until further use. Hematoxylin and eosin (H&E) staining of cryostat-sectioned slides was performed by an automatic staining workstation Tissue Stainer COT 20 (Medite GmbH, Burgdorf, Germany) with standard procedures.

For HIF-1α immunodetection, following blocking with PBS 1% goat serum for 30 minutes, sections were incubated with a mouse monoclonal anti-HIF-1α antibody (Abcam, 1:25) for 16 hours at 4°C in a dark wet chamber. Sections were then washed with bi-distilled water and incubated for 30 minutes at room temperature (RT) with a polymeric secondary antibody conjugated to alkaline phosphatase (AP Histofine Simplestain M). The primary-secondary complex was detected by enzymatic reaction using an appropriate chromogenic substrate (Histofine 415161F, New Fuchsin Substrate Kit) for 5 minutes at RT.

### Immunofluorescence

For immunofluorescence staining, sections were blocked with PBS 2% goat serum 0.3% Triton X-100 for one hour at RT, and then incubated with rabbit monoclonal anti-Ki-67 (Abcam, 1:200), rabbit monoclonal anti-cleaved caspase 3 (Cell Signaling Technology, 1:200), or mouse monoclonal anti-EBP50 (BD Biosciences, 1:50) for one hour at 37°C. After washing in PBS, slides were incubated for one hour at RT with goat anti-mouse or anti-rabbit Alexa Fluor 488- or 546-conjugated antibodies (Invitrogen, 1:800). During the last ten minutes of incubation 4', 6-diamidino-2-phenylindole (DAPI, Invitrogen, 1:100) was added. Sections were analyzed by confocal laser-scanning microscope (Zeiss LSM-710 system, Carl Zeiss Microimaging GmbH) or by fluorescence microscope (Olympus BX61, Olympus Inc.).

### Xenograft generation

*In vivo* experiments were approved by the Basel Cantonal Veterinary Office. NOD/SCID mice, initially obtained by Charles River Laboratories (Sulzfeld, Germany), were bred and maintained under specific pathogen free conditions in the animal facility of the Department of Biomedicine, University of Basel. Eight to ten weeks old mice were used for experiments. For both cell lines, 10^5^ cells were re-suspended in a 1:1 mixture of PBS and Matrigel Matrix Reduced Growth Factor (BD Biosciences) and inoculated subcutaneously (s.c.) into the flanks of recipient mice. Tumor formation was monitored weekly by palpation and caliper measurements. Mice were sacrificed when tumors reached a maximum diameter of 10 mm. Samples from all mice were frozen in RNA Later (Sigma-Aldrich) and embedded in Optimal Cutting Temperature compound (OCT) (CellPath Ltd, UK) for subsequent gene expression evaluation and histological examination after cryosectioning, respectively.

### RNA isolation

Total cellular RNA was extracted from 2D cultured cells, or MCTS at different stages, and tumor tissues from xenograft using the RNeasy^®^ Mini Kit (QIAGEN Ltd., Hombrechtikon, Switzerland) according to the manufacturer's protocol. RNA concentration and quality were determined using Agilent Bioanalyzer 2100 (Agilent Technologies, Santa Clara, CA, USA) or with NanoDrop1 ND-1000 Spectrophotometer (NanoDrop Technologies).

### Quantitative real time PCR

RNA (1μg) was reverse transcribed using M-MLV reverse transcriptase (Invitrogen) following manufacturer's protocol, and cDNA were amplified and analyzed by quantitative real-time PCR (qRT-PCR) using ABI Prism 7300 (Applied Biosystems). Commercially available primer sequences specific for human thymidylate synthase (TYMS) was used (Applied Biosystems). Gene expression was normalized by using human glyceraldehyde-3-phosphate dehydrogenase (GAPDH) housekeeping gene as reference.

### Next generation sequencing and analysis

Non-stranded RNA libraries were prepared by using the Illumina TruSeq sample preparation kit and sequenced on Illumina HiSeq 2000 sequencer at the Quantitative Genomics Facility (QGF) of the Department of Biosystems Science and Engineering (D-BSSE) of the ETH Zurich in Basel.

Single-end RNA-seq reads (50-mers) were mapped to the human genome assembly, version hg19, with SpliceMap ([55], included in the R/Bioconductor package QuasR, version 1.2.2) using the command ‘qAlign(“samples.txt”, “BSgenome.Hsapiens.UCSC.hg19”, splicedAlignment=TRUE)’. Using RefSeq mRNA coordinates from UCSC (genome.ucsc.edu, downloaded in January 2014) and the qCount function, we quantified gene expression as the number of reads that started within any annotated exon of a gene. Nucleotide sequences are deposited in the NCBI at GSE57961. After quality control we excluded the sample 2D.3 from the analysis because of degraded RNA (reads obtained only at the end of transcripts) and poor correlation to other samples. The differentially expressed genes were identified using the edgeR package (version 3.4.2) [56]. Genes with FDR smaller than 0.05 and minimum log2 fold change of 2 were assessed for pathway enrichment analysis in Database for Annotation, Visualization and Integrated Discovery (DAVID) [35, 57].

### Clustering and functional analysis of genes

We performed hierarchical clustering of gene subsets which differed in expression between 2D and Xeno samples (abs log2FC>1) and at the same time did not show differences between 3D3 and Xeno specimens (abs log2FC≤1). Gene clusters obtained were processed using DAVID to identify enriched Gene Ontology (GO) terms. The web server REVIGO (http://revigo.irb.hr/) was used to graphically represent the main gene ontology terms [58]. The clusters are depicted as a Treemap where each rectangle represents a single cluster. Representatives are joined into “superclusters” of loosely related terms, visualized with different colors. Sizes of the rectangles are adjusted to reflect the p-value of the GO term in the underlying GOA database.

### Western blot analysis

Cells lysates were obtained by using a lysis buffer containing 50mM Tris pH 8.0, 150mM NaCl, 1% NP-40, 0.1% SDS or 1mM EDTA, 0.1% Triton X-100, 10mM NaF, 1mM PMSF and 1mM orthovanadate. Protein concentrations were determined by Bradford assay (Bio-Rad Protein Assay; Bio-Rad Laboratories AG, Reinach, Switzerland) and samples were resolved by SDS-PAGE, and transferred onto nitrocellulose membrane (Schleicher & Schuell, Switzerland). Membranes were incubated with primary antibodies against Thymidylate Synthase (1:1000, catalog nr. 9045; Cell Signaling Technology), Thymidine kinase (1:1000, catalog nr. 8960; Cell Signaling Technology), and β-actin (1:2000, A5441; Sigma-Aldrich) diluted in Tris-buffered saline containing Tween-20 (TBST) and 1% bovine serum albumin (BSA) overnight at 4°C. After washes with TBST, membranes were incubated for 1 hour at room temperature with fluorescent secondary goat anti-mouse (IRDye 680) or anti-rabbit (IRDye 800) antibodies (both from LI-COR Biosciences). Blots were analyzed by using the Odyssey Infrared Imaging System (LI-COR Biosciences).

### Drug treatment

The activity of 5-Fluorouracil (5FU; 50mg/mL; Teva Pharma AG, Basel, Switzerland) was tested on HT29 cells cultured in monolayers (7.5*10^3 cells per well) or on spheroids of different sizes, generated as detailed above. Untreated cells and spheroids were cultured in parallel as controls. Different 5FU concentrations were used to represent the wide range of plasma concentrations in patients treated with 5FU [51, 59].

Drug effects on both monolayer and spheroid cultures were recorded after 72h of treatment by acid phosphatase (APH) assay, as described below. All experiments were carried out at least in triplicates. Background correction was performed using the absorbance at 405 nm of the blank sample.

### Acid phosphatase assay

A modified acid phosphatase (APH) assay was used to determine cell viability in spheroids, as previously described [44]. Briefly, 2D cultures and spheroids, previously transferred in flat-bottom 96-well plates, were incubated with substrate solution (0.1M sodium acetate, 0.1% Triton-X-100, supplemented with 2mg/mL of 4-nitrophenyl phosphate disodium; Sigma Aldrich, Saint Louis, MO, USA), mixed 1:1 with PBS (final volume 200μL/well), 90min at 37°C. Following incubation, 10μL of 1N NaOH were added to each well, and absorption at 405nm was measured within 10 minutes on a Synergy H1 Hybrid Multi-Mode Microplate Reader (Biotek Instruments, Luzern, Switzerland).

### Statistical analysis

Data are presented as mean ± standard error. The significance of differences was assessed using analysis of variance (two-way ANOVA) followed by the Tukey's test for multiple comparisons (GraphPad Prism 6). P<0.05 was considered statistically significant.

## SUPPLEMENTARY FIGURES




