# Supplementary material for: Induction of hypoxia and necrosis in multicellular tumor spheroids is associated with resistance to chemotherapy treatment
Source: Oncotarget. 2016 Dec 10;8(1):1725–36. doi: 10.18632/oncotarget.13857 (PMC5352092; doi:10.18632/oncotarget.13857)
Supplement: Supplementary file 1 [file oncotarget-08-1725-s001.pdf]

# Induction of hypoxia and necrosis in multicellular tumor spheroids is associated with resistance to chemotherapy treatment

## SUPPLEMENTARY FIGURES

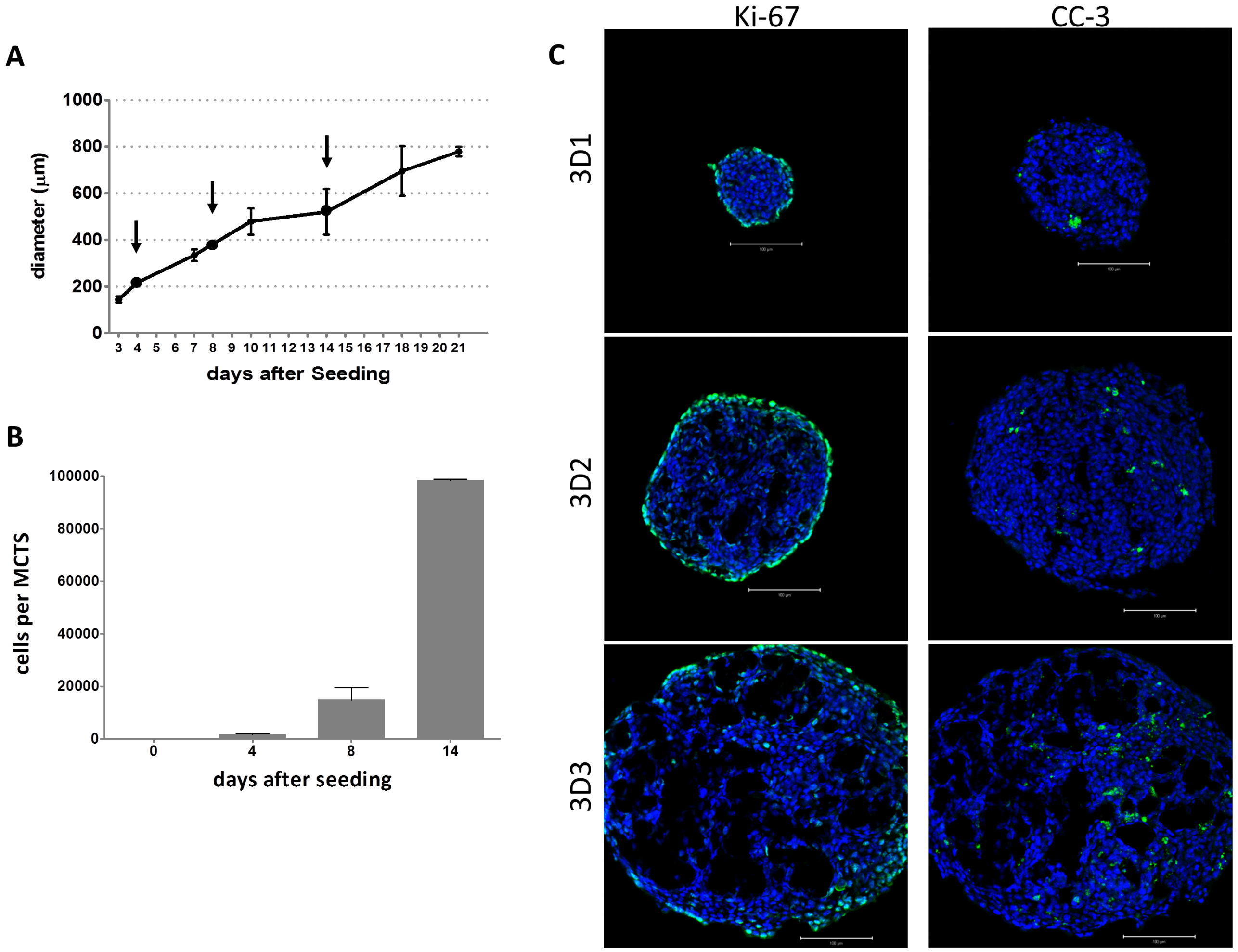


**Supplementary Figure S1: Establishment of HCT116 CRC cell MCTS at different stages.** HCT116 cells were seeded at 100 cells per hanging drop and cultured up to 21 days. MCTS sizes were measured **A.** and cell numbers were counted **B.** at specific time points, as indicated. Ki-67 and CC3 specific staining was performed on sections derived from MCTS at different stages **C.** Magnification 20x; scale bar 100μm.

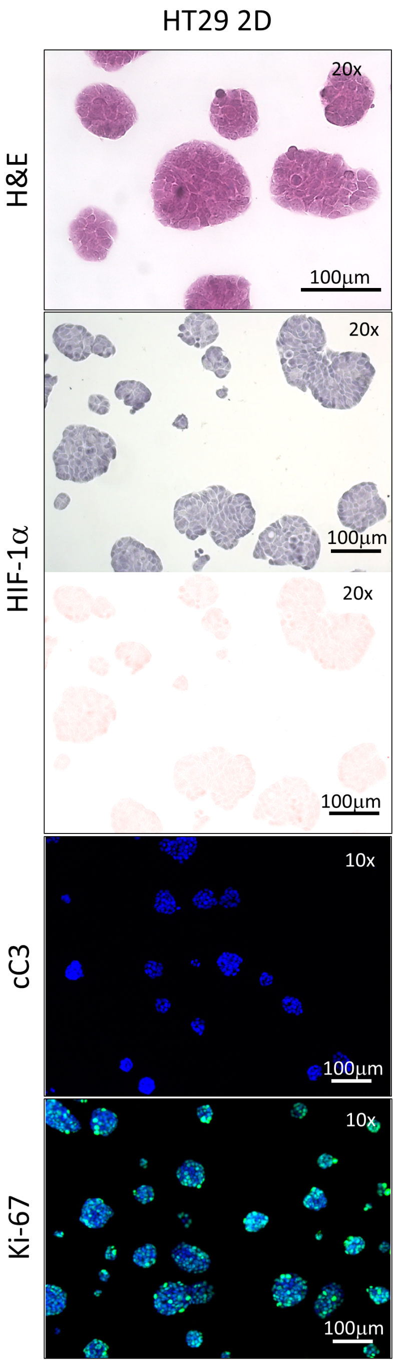


**Supplementary Figure S2: Histological characterization of HT29 cells cultured in 2D.** HT29 cells cultured in 2D were stained for H&E or with anti-HIF1α (upper panel HIF-1a and counterstaining with Hematoxylin, lower panel color deconvolution to show the HIF-1α positivity only), anti-cleaved Caspase 3 (cC3, green) or anti-Ki-67 antibodies (green). In the cC3 and Ki-67 stainings the nuclei were counterstained with DAPI (blue). Magnification 10x and 20x as indicated in the images. Scale bar 100µm as indicated.

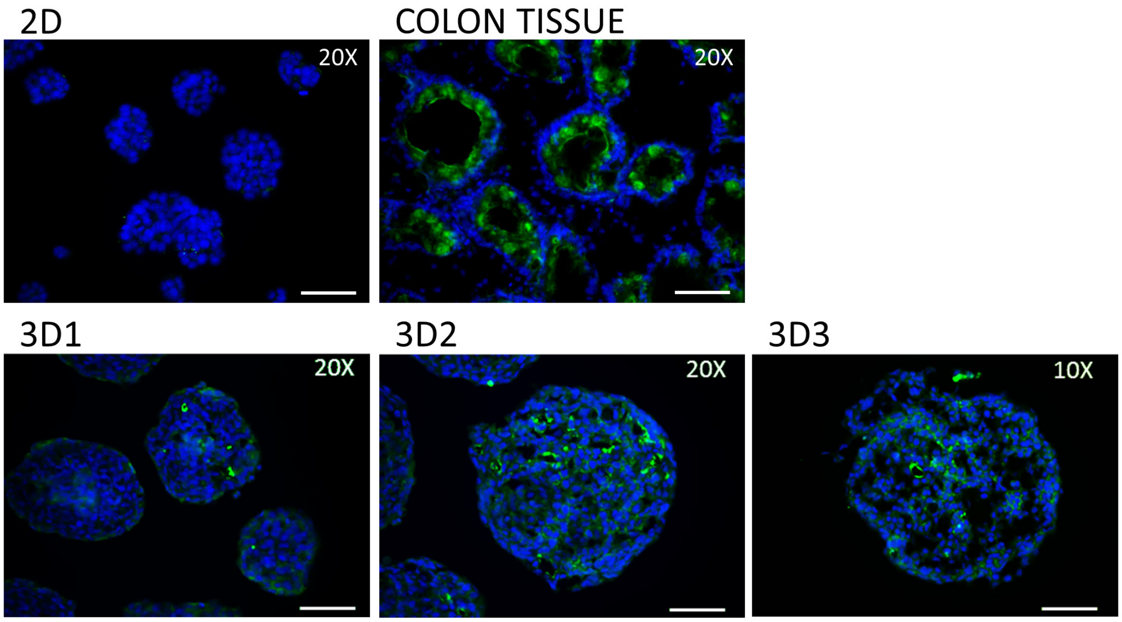


**Supplementary Figure S3: EBP50 specific staining of HT29 CRC cells in different culture conditions.** Cells from healthy colon tissue, HT29 monolayers and HT29 MCTS at different stages were stained with an anti-EBP50 specific antibody. Magnifications 10x and 20x as indicated in the images; scale bar 50µm (3D3 image) and 100µm.

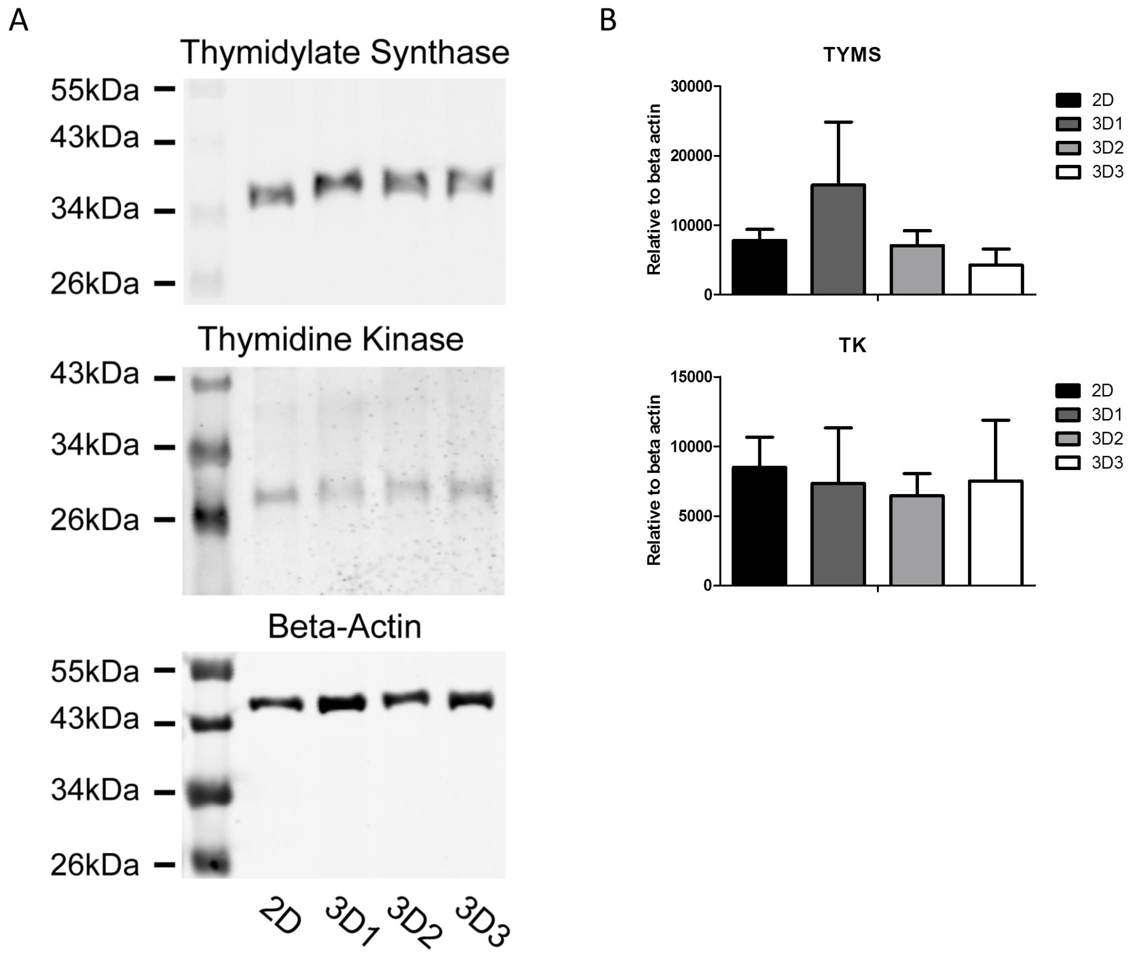


**Supplementary Figure S4: Expression of Thymidylate Synthase and Thymidine Kinase in HT29 cells.** HT29 cells were cultured in monolayers or MCTS of different sizes. Thymidylate synthase (TYMS) and Thymidine kinase (TK) protein expressions were assessed by Western blot analysis using whole-cell lysates panel **A.** Quantifications of TYMS and TK protein amounts relative to Beta-actin are showed as mean and SD from three independent experiments panel **B.**

**Supplementary File S1:**

See Supplementary File 1
